# Breakthrough attacks in patients with hereditary angioedema receiving long-term prophylaxis are responsive to icatibant: findings from the Icatibant Outcome Survey

**DOI:** 10.1186/s13223-017-0203-z

**Published:** 2017-07-05

**Authors:** Werner Aberer, Marcus Maurer, Laurence Bouillet, Andrea Zanichelli, Teresa Caballero, Hilary J. Longhurst, Amandine Perrin, Irmgard Andresen, for the IOS Study Group, W Aberer, M Wiednig, A Grumach, A Bygum, C Blanchard Delauny, I Boccon-Gibod, L Bouillet, B Coppere, O Fain, B Goichot, A Gompel, S Guez, PY Jeandel, G Kanny, D Launay, H Maillard, L Martin, A Masseau, Y Ollivier, A Sobel, J Arnolds, E Aygören-Pürsün, M Baş, M Bauer, K Bork, M Magerl, I Martinez-Saguer, M Maurer, E Papadopoulou-Alataki, F Psarros, Y Graif, S Kivity, A Reshef, E Toubi, F Arcoleo, M Bova, M Cicardi, P Manconi, V Montinaro, G Marone, A Zanichelli, ML Baeza, T Caballero, R Cabañas, M Guilarte, D Hernandez, C Hernando de Larramendi, R Lleonart, T Lobera, L Marques, B Saenz de San Pedro, J Bjoerkander, C Bethune, T Garcez Pereira, M Helbert, HJ Longhurst

**Affiliations:** 1 Department of Dermatology and Venerology, Medical University of Graz, Auenbruggerplatz 8, A-8036 Graz, Austria; 2 Department of Dermatology and Allergy, Allergie-Centrum-Charité, Charité-Universitätsmedizin Berlin, Charitéplatz 1, 10117 Berlin, Germany; 3 National Reference Centre for Angioedema, Internal Medicine Department, Grenoble University Hospital, Boulevard de la Chantourne-CS10217, 38043 Grenoble, France; 4 Department of Biomedical and Clinical Sciences Luigi Sacco, ASST Fatebenefratelli Sacco, University of Milan, Via G.B. Grassi 74, 20157 Milan, Italy; 5 Allergy Department, Hospital La Paz Institute for Health Research (IdiPaz), Biomedical Research Network on Rare Diseases (CIBERER, U754), Paseo de la Castellana 261, 28046 Madrid, Spain; 6 Department of Immunology, Barts Health NHS Trust, 80 Newark Street, London, E1 2ES UK; 7 Shire, Zählerweg 10, 6300 Zug, Switzerland

**Keywords:** Hereditary angioedema, Icatibant, Breakthrough attacks, Prophylaxis, Bradykinin

## Abstract

**Background:**

Patients with hereditary angioedema (HAE) due to C1-inhibitor deficiency (C1-INH-HAE) experience recurrent attacks of cutaneous or submucosal edema that may be frequent and severe; prophylactic treatments can be prescribed to prevent attacks. However, despite the use of long-term prophylaxis (LTP), breakthrough attacks are known to occur. We used data from the Icatibant Outcome Survey (IOS) to evaluate the characteristics of breakthrough attacks and the effectiveness of icatibant as a treatment option.

**Methods:**

Data on LTP use, attacks, and treatments were recorded. Attack characteristics, treatment characteristics, and outcomes (time to treatment, time to resolution, and duration of attack) were compared for attacks that occurred with versus without LTP.

**Results:**

Data on 3228 icatibant-treated attacks from 448 patients with C1-INH-HAE were analyzed; 30.1% of attacks occurred while patients were using LTP. Attack rate, attack severity, and the distribution of attack sites were similar across all types of LTP used, and were comparable to the results found in patients who did not receive LTP. Attacks were successfully treated with icatibant; 82.5% of all breakthrough attacks were treated with a single icatibant injection without C1-INH rescue medication. Treatment outcomes were comparable for breakthrough attacks across all LTP types, and for attacks without LTP.

**Conclusions:**

Patients who use LTP should be aware that breakthrough attacks can occur, and such attacks can be severe. Thus, patients with C1-INH-HAE using LTP should have emergency treatment readily available. Data from IOS show that icatibant is effective for the treatment of breakthrough attacks.

*Trial Registration* NCT01034969

## Background

Hereditary angioedema (HAE) is a rare genetic disorder characterized by recurrent attacks of cutaneous or submucosal edema associated with C1-inhibitor (C1-INH) deficiency (C1-INH-HAE type 1) or dysfunction (C1-INH-HAE type 2), leading to the overproduction of bradykinin, activation of bradykinin B2 receptors, capillary leakage, and local edema [1, 2].

It is recommended that all patients with C1-INH-HAE have access to therapy that can be used to treat acute angioedema attacks (“on-demand treatment”) [3–6]. In addition, patients with C1-INH-HAE may receive long-term prophylaxis (LTP) to reduce the frequency and severity of attacks [5]. However, despite the use of LTP, angioedema attacks, some of them severe, have been reported [7–11]. Therefore, patients receiving LTP also should carry an effective on-demand treatment for acute attacks. The characteristics of attacks occurring during LTP, referred to here as breakthrough attacks, have not been formally examined in depth. Furthermore, there are currently no reports evaluating treatments for breakthrough attacks in detail.

Icatibant (Firazyr^®^, Shire, Zug, Switzerland) is a subcutaneously administered bradykinin B2 receptor antagonist for the treatment of acute angioedema attacks in adults with type 1 or 2 C1-INH-HAE. The Icatibant Outcome Survey (IOS; NCT01034969) is an ongoing, prospective, international, observational study monitoring the safety and effectiveness of icatibant during long-term treatment in the real-world setting. We conducted an analysis of data from IOS to evaluate the characteristics of breakthrough attacks, and compared them with attacks that did not occur during LTP. Outcomes following treatment of these attacks with icatibant also were examined.

## Methods

### Study design and patients

Details for the design and conduct of IOS are described elsewhere [12]. IOS captures real-world data on treatment with icatibant. Enrollment in IOS is open to all patients who have taken at least one dose of icatibant, regardless of attack frequency and level of icatibant usage. Patients with diagnosed C1-INH-HAE were included in this analysis [1]. Data on icatibant-treated attacks and LTP use were collected between July 2009 and February 2016 via physician-completed electronic forms at routine visits (recommended at 6-month intervals), and with the aid of patient diaries. Characteristics of attacks and details regarding treatment with icatibant and any concomitant medications were recorded. Data on attacks and treatments during the 12 months before enrollment are referred to as historical. Attack severity was classified as very mild (very mild interference with daily activities), mild (mild interference with daily activities), moderate (moderate interference with daily activities and no other countermeasures required), severe (severe interference with daily activities with or without other countermeasures), or very severe (very severe interference with daily activities and other countermeasures required).

The type of LTP administered, as well as dose, start and end dates, and frequency of dosing were recorded. Adherence to prophylaxis treatment was not monitored. Attacks that occurred outside a start–end interval were considered to have occurred without LTP; attacks that occurred on the day that LTP started or ended were considered to have occurred with LTP. The total duration of treatment was calculated as the sum of the maximum duration of each type of LTP administered for each patient, irrespective of the occurrence of icatibant-treated attacks. The attack frequency for each type of LTP was calculated by dividing the number of attacks by the total duration of treatment. It was noted that some LTP regimens, such as androgens, had been started years before the availability of icatibant as a treatment option. To avoid underestimating the attack frequency, the date of LTP initiation was replaced with the date of the first treated attack if LTP began before the first treated attack. LTP regimens that started and stopped before the first treated attack were excluded.

Patients received subcutaneous injections of icatibant 30 mg for acute attacks. Icatibant was self-administered (after training by a health care professional), or administered by a health care professional. Many patients self-administered icatibant before the formal approval of this mode of administration in 2011.

### Statistical analyses

Data comparisons were made for attacks occurring with LTP versus without LTP. Attacks with complete data for all three outcomes, (1) time to treatment (defined as time from attack onset to first icatibant injection), (2) time to resolution (defined as time from first icatibant injection to complete symptom resolution of all angioedema), and (3) duration of attack (defined as time from attack onset to complete resolution of symptoms), were compared using a mixed-model analysis of repeated measures (PROC MIXED; SAS Institute Inc., Cary, NC). A generalized linear mixed-model for repeated measures (PROC GLIMMIX) was used to compare the severity of attacks (e.g., very mild/mild/moderate versus severe/very severe).

## Results

### Patient characteristics

Data from 3228 icatibant-treated attacks were analyzed from 448 patients with C1-INH-HAE. Most (94.4%) patients had a diagnosis of C1-INH-HAE type 1 and 58.7% of patients were female. Demographic characteristics of the patients are summarized in Table 1.

**Table 1:** Demographic characteristics

Characteristic	Total
Patients, N	448
HAE diagnosis, n (%)	
Type 1	423 (94.4)
Type 2	25 (5.6)
Sex, n (%)	
Female	263 (58.7)
Male	185 (41.3)
Age at enrollment, y	
Mean (SD)	40.8 (14.7)
Median (min, max)	39.1 (16.5, 81.8)
Country, n (%)	
France	119 (26.6)
Spain	69 (15.4)
Germany	57 (12.7)
United Kingdom	53 (11.8)
Italy	46 (10.3)
Israel	45 (10.0)
Denmark	19 (4.2)
Brazil	16 (3.6)
Greece	14 (3.1)
Austria	8 (1.8)
Sweden	2 (0.4)

*HAE* hereditary angioedema, *max* maximum, *min* minimum

### HAE attacks that occurred with and without LTP shared similar characteristics

Of the 3228 total icatibant-treated attacks recorded, 973 (30.1%) attacks occurred in 171 patients during LTP (38.2% of the total 448 patients in this analysis), and 2255 (69.9%) attacks occurred in 323 patients (72.1%) who either never received LTP (n = 277) or who were not using LTP at the time of the attack (n = 46; Table 2). The analysis included attacks that occurred over a mean (SD) duration of 3.5 (1.8) years per patient on LTP, and 3.6 (1.8) years for patients who were not prescribed LTP (Table 3).

**Table 2:** Characteristics of icatibant-treated attacks in patients with and without LTP

Characteristic	Attacks With LTP	Attacks Without LTP	Total
Patients, n	171	323	448^a^
Attacks, n (%)	973 (30.1)	2255 (69.9)	3228
Attack severity, n (%)^b^			
n^c^	813	2029	2842
Very mild	6 (0.7)	29 (1.4)	35 (1.2)
Mild	65 (8.0)	161 (7.9)	226 (8.0)
Moderate	243 (29.9)	759 (37.4)	1002 (35.3)
Severe	356 (43.8)	803 (39.6)	1159 (40.8)
Very severe	143 (17.6)	277 (13.7)	420 (14.8)
Attack site, n (%)			
n^c^	967	2222	3189
Skin	339 (35.1)	669 (30.1)	1008 (31.6)
Abdomen	460 (47.6)	1158 (52.1)	1618 (50.7)
Larynx	29 (3.0)	110 (5.0)	139 (4.4)
Other organs^d^	23 (2.4)	52 (2.3)	75 (2.4)
Multiple sites	116 (12.0)	233 (10.5)	349 (10.9)
Type of administration, n (%)			
n^c^	923	2153	3076
Self-administration	705 (76.4)	1666 (77.4)	2371 (77.1)
HCP	218 (23.6)	487 (22.6)	705 (22.9)

Analysis included attack data for patients who were or were not receiving LTP at the time of the attack(s) and who were treated with icatibant for acute attacks

*HCP* health care provider, *LTP* long-term prophylaxis

^a^Some patients reported attacks with LTP as well as without LTP

^b^ *P* = 0.147 for comparison of very mild/mild/moderate versus severe/very severe attacks

^c^Excludes attacks with missing or unknown data

^d^Other organs affected by ≥2 attacks include tongue, bladder, esophagus, lungs, respiratory tract, breast, genitals, testicles, bowel, stomach, uvula, joints, and brain

**Table 3:** Duration of treatment and attack frequency for each type of LTP

Type of LTP	No. of attacks (%)	No. of patients (%)	Duration of LTP, y^a^			Attack frequency (attacks/year)^c^
			Mean (SD)	Median (IQR)	Total duration^b^	
C1-INH	121 (12.4)	15 (8.8)	3.4 (1.4)	3.1 (2.6–3.7)	51.5	2.3
C1-INH/androgens^d^	9 (0.9)	4 (2.3)	4.6 (1.4)	4.6 (3.4–5.8)	18.6	0.5
C1-INH/androgens/tranexamic acid	4 (0.4)	2 (1.2)	3.3 (1.9)	3.3 (1.9–4.6)	6.5	0.6
C1-INH/tranexamic acid	24 (2.5)	2 (1.2)	3.8 (1.1)	3.8 (3.1–4.6)	7.7	3.1
Androgens	542 (55.7)	108 (63.2)	3.5 (1.9)	3.4 (2.2–5.0)	372.7	1.5
Androgens/tranexamic acid	41 (4.2)	9 (5.3)	5.0 (1.3)	4.5 (4.1–6.0)	44.8	0.9
Tranexamic acid	232 (23.8)	43 (25.1)	3.4 (1.8)	3.8 (1.6–4.6)	147.1	1.6
Overall	973	171	3.5 (1.8)	3.6 (2.2–4.8)	620.5	1.6
No LTP^e^	1941	277	3.6 (1.8)	3.4 (2.1–5.0)	985.4	2.0

*C1*-*INH* C1-inhibitor, *IQR* interquartile range, *LTP* long-term prophylaxis

^a^The start date of LTP was imputed to the date of the first treated attack if the patient started LTP before the date of the first attack

^b^The sum of maximum treatment duration. Treatments received by patients were included even if they were not related to treated attacks

^c^Icatibant-treated attacks that occurred with or without LTP

^d^Androgens include danazol, stanozolol, and oxandrolone

^e^The duration (follow-up time) of icatibant treatment for patients who never received LTP. Duration was computed from first attack date to extract date, death date, or discontinuation date, as applicable. Patients who reported attacks with and without LTP were excluded

Androgens (danazol, oxandrolone, or stanozolol) were the most commonly used form of LTP. Androgens were used by 108/171 (63.2%) patients, while 43 (25.1%) patients used tranexamic acid and 15 (8.8%) patients used C1-INH. In addition, 17 (9.9%) patients used various combinations of these three medications. The mean (SD) duration of use was 3.5 (1.9), 3.4 (1.8), and 3.4 (1.4) years for androgens, tranexamic acid, and C1-INH, respectively. The rate of icatibant-treated breakthrough attacks was similar irrespective of the type of prophylaxis; there were 1.5, 1.6, and 2.3 attacks/year reported in patients using androgens, tranexamic acid, and C1-INH for LTP, respectively. In comparison, there were 2.0 attacks/year in patients who never received LTP.

**Fig. 1:**
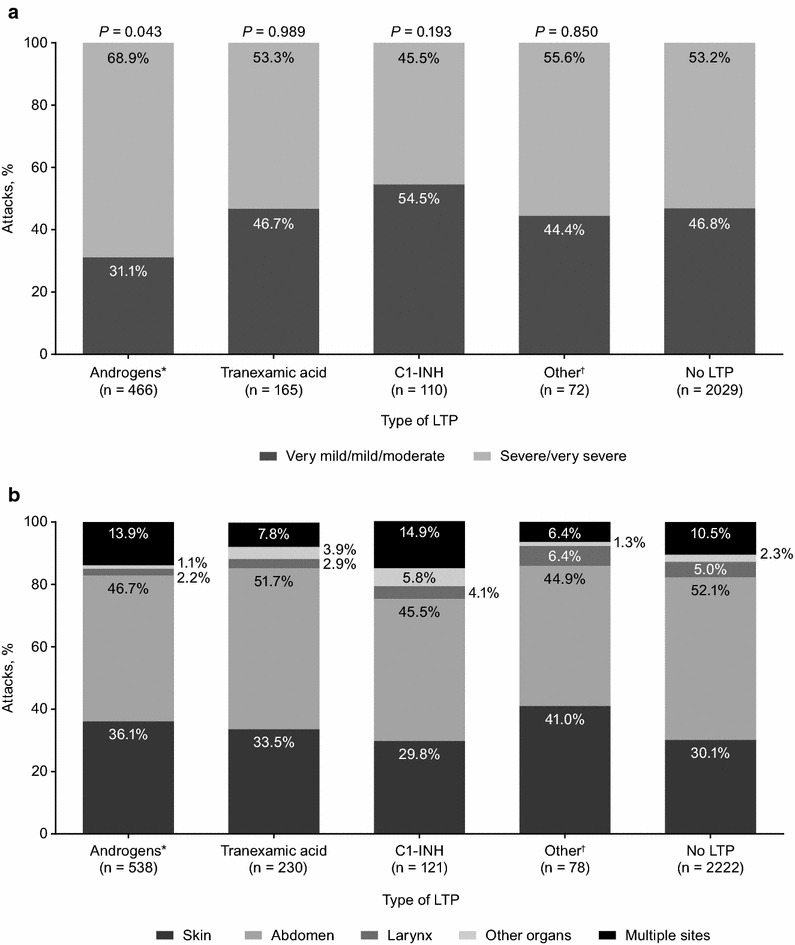
**a** Severity and **b** site of attacks by type of long-term prophylaxis (LTP). *P* values compared severity of attacks with LTP versus attacks without LTP. Attacks with **a** unknown severity and **b** unknown attack site were excluded. *Androgens include danazol, stanozolol, and oxandrolone. ^†^Other includes all LTP treatments that combined >1 type of LTP

There were no statistically significant differences in attack severity in patients who used androgens, C1-INH, or tranexamic acid for LTP compared with attacks that occurred without LTP (Fig. 1a). In patients who used androgens for LTP, 68.9% of attacks were severe/very severe and 31.1% of attacks were very mild/mild/moderate compared with 53.2 and 46.8%, respectively, in attacks without LTP (*P* = 0.056). The proportions of severe/very severe and very mild/mild/moderate attacks were comparable between patients receiving C1-INH or tranexamic acid and were similar to those without LTP (*P* = 0.321 and *P* = 0.989 for C1-INH and tranexamic acid, respectively, versus patients without LTP).

The most frequent site of attack was the abdomen (47.6% with and 52.1% without LTP), followed by the skin (35.1 and 30.1%, respectively), and larynx (3.0 and 5.0%, respectively; Fig. 1b). Multiple sites were affected in 12.0% of attacks with LTP and 10.5% of attacks without LTP. The distribution of attack sites was comparable across the different types of LTP. Of the attacks involving the larynx or abdomen, 63.6 and 73.9%, respectively, were severe/very severe.

### Most attacks with LTP were successfully treated with a single icatibant injection

**Fig. 2:**
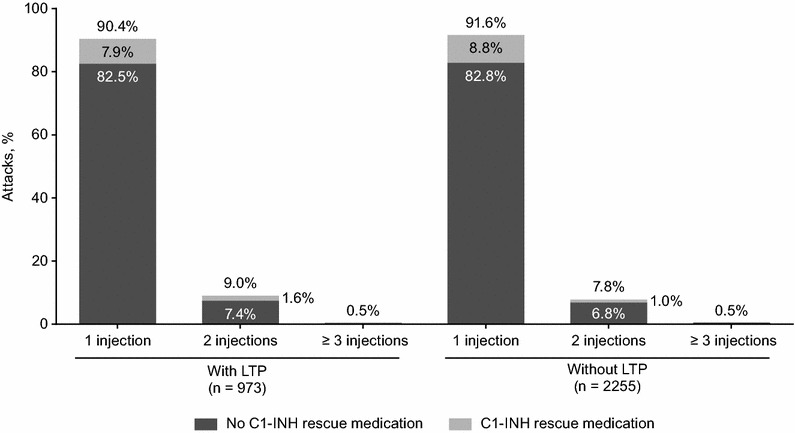
Proportion of attacks treated with 1, 2, or ≥3 injections of icatibant and C1-inhibitor (C1-INH) rescue medication(s) in patients who received or did not receive long-term prophylaxis (LTP)

Icatibant was self-administered in 76.4% of breakthrough attacks and in 77.4% of attacks that occurred without LTP. Overall, 803/973 (82.5%) breakthrough attacks and 1868/2255 (82.8%) attacks without LTP were treated with a single dose of icatibant, and without the use of C1-INH rescue medication (Fig. 2). Multiple icatibant injections were used in 93 (9.6%) breakthrough attacks and in 189 (8.4%) attacks that occurred without LTP. C1-INH rescue medication was used in 95 (9.8%) breakthrough attacks and in 221 (9.8%) attacks that occurred without LTP. Patients also reported using other medications in addition to icatibant, the most frequent being androgens, analgesics, and antifibrinolytics. These were used in addition to icatibant in 3.5, 2.4, and 2.8% of breakthrough attacks, respectively, and in 1.0, 1.6, and 1.2% of attacks without LTP, respectively.

### Outcomes of icatibant-treated HAE attacks were similar for attacks with and without LTP

The median times to treatment with icatibant, time to resolution, and duration of attack were not significantly different for attacks that occurred with versus without LTP (Table 4). Among the different types of LTP, only the combined treatments (“other”) had a shorter time to treatment (*P* = 0.029). There was no significant difference (*P* > 0.100) among the different types of LTP with respect to time to resolution. Duration of attack was significantly longer for breakthrough attacks that occurred while using tranexamic acid (*P* = 0.016), and shorter for attacks with C1-INH (*P* = 0.041). Of note, patients who used tranexamic acid for LTP also had longer time to treatment and longer time to resolution compared with patients who used other types of LTP.

**Table 4:** Time to treatment, time to resolution, and duration of attack for icatibant-treated breakthrough attacks by type of LTP

	Type of LTP					
	LTP	Androgens^a^	Tranexamic acid	C1-INH	Other^b^	No LTP
No. patients	93	56	24	5	9	179
No. attacks^c^	391	226	92	36	37	781
Time to treatment						
Median (IQR), h	1.5 (0.5–4.0)	1.3 (0.5–4.0)	2.0 (0.5–5.9)	1.5 (0.1–2.1)	0.8 (0.5–2.0)	1.0 (0.3–4.0)
*P* value^d^	0.090	0.095	0.745	0.681	0.029	
Time to resolution						
Median (IQR), h	4.5 (2.0–12.0)	5.0 (2.0–11.5)	7.0 (2.0–20.5)	3.0 (2.4–4.0)	4.0 (2.0–24.5)	6.0 (2.0–14.6)
*P* value	0.869	0.642	0.317	0.103	0.565	
Duration of attack						
Median (IQR), h	8.0 (4.0–17.0)	8.0 (3.5–17.0)	11.6 (6.0–25.3)	4.0 (3.1–5.8)	7.0 (3.0–25.0)	9.0 (3.8–20.0)
*P* value	0.543	0.984	0.016	0.041	0.741	

*C1*-*INH* C1-inhibitor, *IQR* interquartile range, *LTP* long-term prophylaxis

^a^Androgens include danazol, stanozolol, and oxandrolone

^b^Other includes all LTP treatments that combined >1 type of LTP

^c^Only attacks with complete data for all outcomes were included in this analysis

^d^ *P* value derived from a mixed model of repeated measures and compares attacks with versus without LTP

Two patients were previously identified as outliers based on their reinjection characteristics [13]. These patients used LTP (danazol and C1-INH, respectively) during enrollment in the study. The results for icatibant reinjection and treatment outcomes between attacks that occurred with or without LTP were not significantly impacted when data from these outliers were excluded.

## Discussion

Prophylactic treatment for C1-INH-HAE has been shown to reduce the frequency and severity of attacks [7, 14, 15]. The present analyses of real-world data from IOS support that patients receiving LTP (who usually have high disease activity) experience attacks of similar duration and frequency as patients who do not use LTP. Our findings show that icatibant, when used as on-demand medication, is similarly effective in controlling attacks in patients who are taking LTP and in those who are not.

In our dataset, patients across all LTP groups experienced breakthrough attacks, including those that involved the larynx and those that significantly impaired physical function. Breakthrough attacks may have occurred because local C1-INH levels fell below or did not reach the normal range despite administration of LTP, resulting in inadequate regulation of the contact system [1]. It is possible that doses of LTP administered were insufficient, or the dosing was not frequent enough to completely prevent attacks. In addition, data regarding patient adherence to LTP were not available, and breakthrough attacks may have resulted from delays in dosing, or errors in the administration of prophylactic medications.

Patients who are candidates for LTP typically have more severe disease (i.e., frequent and severe attacks) [1, 3, 4]. Approximately half of the attacks reported in patients taking LTP were severe/very severe, which is comparable to attacks that occurred without LTP. This further emphasizes the fact that breakthrough attacks can occur often and can be severe. Interestingly, there was a higher proportion of severe/very severe attacks in patients who were using androgens for LTP (68.9%) compared with patients who were using tranexamic acid, C1-INH, or a combination of the three treatments (45.5–55.6%). However, we cannot make conclusions regarding efficacy from this observation as the severity of disease prior to starting LTP among the patients who used the various types of LTP is not known. Furthermore, androgens are associated with numerous side effects [16], which may compromise patient compliance and subsequently treatment effectiveness.

Irrespective of LTP use, patients responded well to icatibant, and >80% of C1-INH-HAE attacks were successfully treated with only a single injection of icatibant. This is consistent with previous results reported from IOS [13, 17]. The rate of on-demand medication use was comparable for attacks that occurred with or without LTP, and there was no difference in treatment outcomes between attacks that occurred with or without LTP. Duration of attack was longer for attacks that occurred while taking tranexamic acid, and shorter with C1-INH treatment, in accordance with previous reports [14, 18]. Statistically significant effects of individual types of LTP on time to treatment and duration of attack should be interpreted with caution in this analysis, however, as both the number of patients and frequency of attacks within these groups were small.

Overall, the findings from IOS indicate that attack characteristics and outcomes with icatibant treatment were similar for patients with high disease activity (i.e., those who require LTP treatment), and for those with less severe disease. These results are consistent with our clinical experience with icatibant treatment in patients with C1-INH-HAE. Furthermore, the results support the theory that attacks in patients with high- and low-disease severity share a common pathogenic mechanism [19]. Specifically, this involves elevation of bradykinin levels subsequent to contact system activation in the absence of sufficient, protective C1-INH levels; icatibant counteracts this activity by inhibiting the bradykinin receptor.

The results also revealed some surprising information concerning the use of icatibant plus add-on medications to treat attacks. Although attenuated androgens and antifibrinolytics are not recommended for the treatment of acute attacks [4], approximately 3% of total attacks were treated with these medications following dosing with icatibant. Of the 56 attacks that were treated with icatibant plus on-demand androgens, 34 (60.7%) attacks occurred while androgens were also used for LTP, suggesting that if icatibant were determined to be insufficient for resolving an attack, patients simply resorted to readily available treatments for rescue. The remaining 22 attacks that were treated with on-demand androgens occurred without LTP; these attacks were reported by 11 patients, and may reflect a lack of knowledge on the part of the patients or physicians regarding selection of appropriate treatments for acute attacks. Androgens might also be used as a form of short-term prophylaxis to reduce the chance of occurrence of a later attack (for example prior to traveling or a dental procedure).

Some limitations of this study should be noted. Although the data suggest that the attack rate while using C1-INH, androgens, or tranexamic acid for LTP was similar and that LTP was effective in reducing the attack rate of patients with severe disease to the same rate as patients with less severe disease, this was not a randomized trial in which patients were randomly assigned to receive LTP, and adherence to LTP treatment was not monitored. The goal of this analysis was to evaluate the effectiveness of icatibant in treating breakthrough attacks, rather than to compare different LTP options. Data confirming disease severity prior to initiation of prophylactic treatment were also not available as a baseline comparison. In addition, the C1-INH treatment group was relatively small. Thus, these data cannot be used to evaluate the effectiveness of LTP or to compare the various LTP treatments. The small number of C1-INH users in the database population does reflect our clinical experience in that very few patients use C1-INH at the recommended dose of 1000 U every 3–4 days; instead, doses and regimens are often adjusted according to individual patients’ responses. Thus it is difficult to fully assess the impact of C1-INH in comparison to other LTP treatments. In addition, C1-INH is indicated for both LTP and the treatment of acute attacks, and patients who use C1-INH for LTP often use it to treat breakthrough attacks as well [20]. As this analysis only includes attacks treated with icatibant, it is possible that some attacks were treated with C1-INH or other medications instead of icatibant, thus the true number of attacks with and without LTP could potentially be higher than reported here. Information on attacks that were treated with medications other than icatibant is not included in IOS. Of the 121 breakthrough attacks that occurred while on prophylaxis with C1-INH, 20 attacks were treated with rescue medication, and C1-INH was used exclusively for all 20 attacks. This further emphasizes the high frequency of C1-INH use for both prophylaxis and acute treatment indications.

## Conclusions

Icatibant was successfully used to treat both breakthrough attacks in patients with C1-INH-HAE receiving prophylaxis as well as attacks that occurred without LTP. Patients who are prescribed LTP should be aware of the potential for breakthrough attacks, including those of high severity and involving the larynx, and should be prepared by having easily accessible emergency treatment.

## Abbreviations

C1-INH: C1-inhibitor
C1-INH-HAE: hereditary angioedema due to C1-inhibitor deficiency
HAE: hereditary angioedema
HCP: health care provider
IOS: Icatibant Outcome Survey
IQR: interquartile range
LTP: long-term prophylaxis
